# A retrospective review of speech-language therapy services provided to adult inpatients at a central-level hospital in Gauteng, South Africa

**DOI:** 10.4102/sajcd.v67i1.707

**Published:** 2020-11-26

**Authors:** Jennifer Stone, Azra Hoosen, Hayley Hochfelden, Innocent Maposa, Shajila Singh

**Affiliations:** 1 Department of Speech Therapy and Audiology, Chris Hani Baragwanath Academic Hospital, Johannesburg, South Africa; 2 Division of Epidemiology and Biostatistics, University of the Witwatersrand, Johannesburg, South Africa; 3 Department of Communication Sciences and Disorders, University of Cape Town, Cape Town, South Africa

**Keywords:** speech-language therapy, dysphagia, aphasia, dysarthria, intervention, burden of disease, multimorbidity, public healthcare

## Abstract

**Background:**

The quadruple burden of disease (BoD) and multimorbidity reflected in South Africa’s public health sector challenges speech-language therapists (SLTs) to optimise patient management in this context. For planning and delivery of appropriate services, it is important to understand the profile of speech-language therapy (SLT) patients and the public healthcare services provided by SLTs.

**Objectives:**

This study aimed to describe the prevalence of inpatient adult speech, language and swallowing disorders associated with various medical conditions and South Africa’s BoD, in addition to the target areas and duration of SLT interventions provided at a central public hospital.

**Method:**

A retrospective review was conducted on records of 2549 adult inpatients who received SLT services between January 2014 and December 2015 at Chris Hani Baragwanath Academic Hospital. Data, including demographics, medical and SLT diagnoses, and treatment recommendations, were analysed using descriptive and inferential statistics.

**Results:**

Non-communicable diseases (NCDs) were most prevalent (77.48%), with multimorbidity of BoD categories in 29.27% of patients. Cerebrovascular disease (CeVD) comprised 52.45% patients, with CeVD, traumatic brain injury, other neurological conditions, cancer and burns comprising 88.74% patients. More than a third of the patients with CeVD were < 56 years (*n* = 486; 36.35%). Dysphagia (48.96%), aphasia (30.95%) and dysarthria (23.62%) were the most common, with 44.68% of patients having multiple SLT diagnoses. The number of SLT sessions significantly correlated with SLT comorbidity (*r*_s_ = 0.4200; *p* = 0.0000), but not BoD comorbidity (*r*_s_ = 0.0049; *p* = 0.8058).

**Conclusion:**

Speech-language therapy patients reflected a heavy NCD burden and multimorbidity. Provision of SLT services should take into consideration a profile of increased complexity of medical conditions and SLT diagnoses.

## Introduction

South Africa’s health system operates on two tiers, the private and the public health sectors, which have been described as divided along socio-economic lines (Young, 2016). In 2015, 55.5% of South Africans were recorded as living in poverty (Statistics South Africa [STATS SA] 2017a). This has been associated with poorer education, income, health insurance and employment (Baugh et al., 2019) and describes many of the users of South Africa’s public health sector. The public health sector, which serves approximately 48 million people (83.6%) (STATS SA, 2018a), is challenged by a quadruple burden of disease (BoD), namely, communicable, maternal and perinatal, non-communicable and injury-related disorders (Pillay-Van Wyk et al., 2016). Although human immunodeficiency virus (HIV) and acquired immunodeficiency syndrome (AIDS) remain among the leading causes of death in South Africa (Pillay-Van Wyk et al., 2016; Stats SA: P0309.3, 2018), the roll-out of antiretroviral treatment has contributed to a 52.6% reduction in HIV-related mortality since 2007 (STATS SA, 2019). This has shifted the country’s BoD profile from HIV and AIDS to multimorbidity (the presence of two or more medical conditions) and mortality (Arokiasamy et al., 2015; Folb et al., 2015) because of non-communicable diseases (NCDs).

Human immunodeficiency virus and hypertension are amongst the leading risk factors for cerebrovascular disease (CeVD) in sub-Saharan Africa (Namale et al., 2018), with CeVD listed among the five leading causes of death (STATS SA, 2018b). Globally, CeVD mortality is declining while the incidence amongst young and middle-aged adults is rising (Feigin et al., 2017; Onaolapo, Onaolapo, & Nathaniel, 2019). This has been noted to be especially true of low- and middle-income economies and raises concerns regarding the effect on social, emotional and economic well-being of young people with CeVD (Onaolapo et al., 2019). The increase in CeVD amongst younger adults has been attributed to a rise in traditional risk factors such as hypertension, diabetes, obesity, dyslipidaemia and smoking in the population in the United States, China and Estonia (George, 2020; Guan et al., 2017; Onaolapo et al., 2019; Schneider, Kornejeva, Vibo, & Kõrv, 2017). Hoffmann (2000) reported HIV as an increasing risk of CeVD in younger South African adults; however, more recent data from African countries have not been reported. It is, therefore, important to explore the profile of such patients relative to the current BoD in South Africa.

Few international and local studies describe the prevalence of speech, language and swallowing disorders across the broad range of medical conditions. Stipancic, Borders, Brates and Thibeault (2019) reported on the incidence of dysphagia (32%), dysarthria (26%) and aphasia (16%) post-CeVD, with comorbid communication diagnoses reported for as many as 69% of those presenting with dysphagia, alluding to the complexity of patients to be managed by speech-language therapists (SLTs).

Only limited data are available regarding speech-language therapy (SLT) pathologies and services in South Africa (Moonsamy, Mupawose, Seedat, Mophosho, & Pillay, 2017; Overett & Kathard, 2006). Furthermore, the impact of the changing BoD profile on SLT service provision to adults in South African public hospitals has not been reported. It is critical for healthcare professionals continuously to optimise the effectiveness and efficiency of rehabilitation services (Winstein et al., 2016), particularly in the context of the evolving case demographics and the multimorbidity reflected in the national BoD. Designing SLT services that are accessible, relevant and responsive to the local BoD requires an understanding of the current patterns of SLT service provision (Pillay-Van Wyk et al., 2016). The study aimed to describe the prevalence of speech, language and swallowing disorders associated with various medical conditions (e.g. CeVD, traumatic brain injury [TBI]) in relation to South Africa’s BoD at the country’s largest public-sector hospital. The care pathways, referrals, target areas and duration of SLT interventions as well as the status at end of service were also described.

## Methods

### Participants

Following approval by the Human Research Ethics Committee at the University of Cape Town (HREC488/2016) and permission from the hospital’s management and Medical Advisory Committee, a retrospective study was conducted using the electronic records of all adult (≥ 18 years) inpatients at Chris Hani Baragwanath Academic Hospital, who had received SLT services between January 2014 and December 2015. In all, 35 patients were excluded, where the SLT services commenced or ended outside of the study time frame. A total of 2549 patients were included.

### Instrument

An electronic spreadsheet with a closed list of response options for each variable was used to abstract data from an existing database of SLT records maintained by SLTs who managed patients during the study time frame. Data abstracted included participant characteristics (e.g. age and sex); medical conditions; SLT diagnoses; treatment recommendations; referral source; mortality; number of days between admission, referral, initial SLT contact and end of service; number of SLT sessions (including all patient contacts for screening, assessment, management and counselling) and the status at end of service.

### Procedure

Following data abstraction, HIV and TB status were verified for each patient by accessing the electronic National Health Laboratory Service database. Data were consolidated by assigning patients presenting with maternal conditions (*n* = 15; 0.59%) to another BoD category according to the medical condition. Inter-rater reliability was addressed by two researchers reviewing and recapturing the data of 260 (10.2%) randomly selected records blinded to the original data set. Agreement was calculated using Cohen’s kappa (*κ*) and was found to be moderate for SLT recommendations (κ = 0.6538), status at the end of inpatient SLT service (*κ* = 0.7104), SLT diagnosis (κ = 0.7616) and medical condition (*κ* = 0.7565) and almost perfect for referral source (*κ* = 0.9186) (McHugh, 2012). Pearson’s rho (*ρ*) was used to determine the inter-rater reliability for the number of SLT sessions recorded and was found to be very strong (*r*_s_ = 0.965) for this variable (Prion & Haerling, 2014).

### Data analysis

Categorical variables were reported as frequency and percentage. Continuous variables were presented as mean and standard deviation (SD) or as median and interquartile range (IQR). A *t*-test for normally distributed data, or Mann–Whitney *U*-test for non-normally distributed data, was used to compare the differences in continuous variables. Cohen’s *κ* and Pearson’s (*r*_s_) correlation coefficients were used to determine the inter-rater agreement. Spearman’s correlation coefficients were used to assess the strength of relationships between continuous variables that were either normally or non-normally distributed. Analysis of multiple response variables considered all possible responses, resulting in cumulative percentage totals > 100 in some analyses (Tables 1–3 and 7). The CeVD risk factors were determined using bivariate logistic regression analysis and were presented as adjusted multivariable odds ratios (aORs) and 95% confidence intervals. In the cases where data were missing (one case where age had not been captured and two cases where the date of admission was not available), complete case analysis was used.

**TABLE 1:** Burden of disease profile (*n* = 2549).

Burden of disease	*n*§	%
1. Communicable diseases:	688	26.99†
HIV:		
Confirmed	622	24.40
Unconfirmed	1158	45.43
Negative	769	30.17
TB:		
Confirmed	248	9.73
Unconfirmed	1852	72.66
Negative	449	17.61
Others:		
Confirmed	119	4.67
2. Non-communicable disease	1975	77.48
3. Injuries	602	23.62
4. None‡	8	0.31

† , Patients in whom at least one communicable disease was confirmed;

‡ , Patients in whom the medical condition was unconfirmed;

§ , Due to the presentation of comorbidities, the total number of medical conditions is greater than the number of patients.

HIV, human immunodeficiency virus; TB, Tuberculosis.

**TABLE 2:** Medical conditions (*n* = 2549).

Medical conditions	*n*§	%
1. Cerebrovascular disease	1337	52.45
2. Traumatic brain injury	317	12.44
3. Other neurological†	281	11.02
4. Cancer	182	7.14
5. Burns	145	5.69
6. Unknown/unspecified	120	4.71
7. Seizures	118	4.63
8. Tracheostomy	105	4.12
9. Meningitis	82	3.22
10. Neurodegenerative disorder	69	2.71
11. Facial/neck trauma (excluding TBI)	64	2.51
12. Other trauma‡	63	2.47
13. HIV related	27	1.06
14. Psychiatric disorder	24	0.94
15. Hydrocephalus	18	0.71
16. Voice disorder	11	0.43
17. Audiological difficulty	11	0.43
18. Structural abnormality¶	10	0.39
19. Intellectual impairment	5	0.20
20. Congenital syndrome	1	0.04

† , e.g. Myasthenia gravis, Guillain–Barré syndrome and ring-enhancing lesions;

‡ , Excluding TBI and facial/neck trauma (e.g. stab to abdomen);

§ , Due to the presentation of comorbidities, the total number of medical conditions is greater than the number of patients;

¶ , Predominantly oesophageal/subglottic stenosis/stricture of various aetiology.

TBI, traumatic brain injury; HIV, human immunodeficiency virus.

**TABLE 3:** Speech-language therapy diagnosis (*n* = 2549).

SLT diagnosis	*n*‡	%
1. Swallowing disorder	1248	48.96
2. Speech disorder:	1139	44.68
Dysarthria	602	23.62
Apraxia	223	8.75
Voice disorder	212	8.32
Burns: Risk of contractures	97	3.81
Dysfluency	5	0.20
3. Language disorder:	849	33.31
Aphasia	789	30.95
Right hemisphere language disorder	60	2.35
4. Other:	479	18.79
Hearing loss	30	1.18
Inconclusive	221	8.67
Others†	78	3.06
Unresponsive	150	5.88
5. Cognitive-communication disorder	279	10.95
6. None	234	9.18

† , Diagnosis could not be categorised (e.g. temporary intubation requiring augmentative and alternative communication [AAC]; food refusal; general medical weakness affecting function);

‡ , Due to the presentation of comorbidities, the total number of SLT diagnoses is greater than the number of patients.

SLT, Speech-language therapy.

### Ethical consideration

This study was approved by the Human Research Ethics Committee at Cape Town University (HREC488/2016). Approval was also granted by the Medical Advisory Committee and hospital management at Chris Hani Baragwanath Academic Hospital in Soweto, Johannesburg, where the study was conducted.

## Results

Results are presented in accordance with the aims and objectives of this study.

### Case profile

#### Burden of disease and medical conditions

The study participants were composed of 1363 males (53.47%) and 1186 females (46.53%). Non-communicable diseases presented most frequently (*n* = 1975; 77.48%) (Table 1). Cerebrovascular disease was the most frequent medical condition (*n* = 1337; 52.45%) (Table 2); and of the patients with CeVD, 117 (8.75%) were 18–25 years of age, 369 (27.60%) were 26–55 years and 851 (63.65%) were >56 years. When controlling for age and sex, hypertension was significantly correlated with CeVD (aOR = 4.82; *p* < 0.001), while diabetes mellitus was not (aOR = 0.91; *p* < 0.590). HIV significantly correlated with CeVD (aOR = 1.44; *p* = 0.004), however, HIV status was unknown in 45.43% of patients. Almost a third (*n* = 746, 29.27%) of the patients had multiple medical conditions across more than one BoD category (Table 4).

**TABLE 4:** Co-occurring burden of disease categories and number of speech-language therapy sessions.

Number of burden of disease categories	*n*	%	Number of SLT sessions								
			Median	IQR	Mean	Standard deviation	Standard error	95% confidence interval for mean		Minimum	Maximum
								Lower bound	Upper bound		
0	8	0.31	1	1–2	3.38	5.93	2.10	0.00	8.33	1	18
1	1795	70.42	4	2–7	5.59	5.11	0.12	5.36	5.83	1	46
2	679	26.64	4	2–7	5.86	5.68	0.22	5.43	6.29	1	37
3	67	2.63	4	2–8	5.76	5.02	0.61	4.54	6.99	1	21
Total	2549	100	4	2–7	5.66	5.27	0.10	5.46	5.87	1	46

SLT, speech-language therapy; IQR, interquartile range.

#### Speech-language therapy diagnoses

Overall, dysphagia (*n* = 1248, 48.96%), aphasia (*n* = 789, 30.95%) and dysarthria (*n* = 602, 23.62%) were the most frequent SLT diagnoses (Table 3), with 44.68% of patients (*n* = 1139) presenting with multiple co-existing SLT diagnoses (Table 5).

**TABLE 5:** Co-occurring speech-language therapy diagnoses and number of speech-language therapy sessions.

Number of SLT diagnoses	*n*	%	Number of SLT sessions								
			Median	IQR	Mean	Standard deviation	Standard error	95% confidence interval for mean		Minimum	Maximum
								Lower bound	Upper bound		
0	552	21.65	2	1–4	2.95	3.15	0.13	2.69	3.21	1	26
1	858	33.66	4	2–7	5.28	4.59	0.16	4.97	5.59	1	46
2	772	30.29	5	3–8	6.61	5.51	0.20	6.22	7.00	1	39
3	357	14.01	6	4–11	8.44	6.34	0.34	7.78	9.10	1	37
4	10	0.39	12	9–24	15.70	8.93	2.83	9.31	22.09	7	32
Total	2549	100	4	2–7	5.66	5.27	0.10	5.46	5.87	1	46

SLT, speech-language therapy; IQR, interquartile range.

### Care pathway

#### Referrals

Doctors referred the highest number of patients for SLT services (*n* = 1514; 59.40%). Referrals from physiotherapists (*n* = 279; 10.95%), occupational therapists (*n* = 207; 8.12%), dietitians (*n* = 115; 4.51%), nurses (*n* = 11; 0.43%) and social workers (*n* = 1; 0.04%) contributed to 24.05% of the caseload (*n* = 613). Speech-language therapists identified patients by means of screening in the wards (*n* = 411; 16.12%) and patients were also referred into the SLT service by patients’ friends or relatives, the patients themselves or patients within the same ward (*n* = 11; 0.43%). Overall, patients presenting with dysphagia (*n* = 1107; 43.43%), aphasia (*n* = 648; 25.42%) and dysarthria (*n* = 516; 20.24%) were the most frequently referred.

#### Time frames

The correlation between the number of days from admission to referral for SLT services (median = 4; IQR = 1–2) and the number of days from initial SLT contact to the end of SLT service (median = 6; IQR = 2–12) (Table 6) was not significant (*r*_s_ = 0.014; *p* = 0.469). The number of SLT sessions (mean = 5.66; SD = 5.27) (Tables 4 and 5) was not correlated with comorbidities across BoD categories (*r*_s_ = 0.0049; *p* = 0.8058) but was significantly correlated with comorbid SLT diagnoses (*r*_s_ = 0.4200; *p* = 0.0000).

**TABLE 6:** Days between admission, referral, initial speech-language therapy contact and end of speech-language therapy service.

Number of days	*n*	Minimum	Maximum	Median	IQR	Mean	Standard deviation
Admission to referral	2547	0	141	4	2–10	8.89	13.07
Referral to initial SLT contact	2549	0	9	0	0–0	0.25	0.72
Initial SLT contact to end of SLT service	2549	0	128	6	2–12	9.16	11.25
Admission to end of SLT service	2547	0	144	13	7–23	18.32	17.31

SLT, speech-language therapy; IQR, interquartile range.

#### Speech-language therapy treatment recommendations

Overall, recommendations for dysphagia interventions were most frequent (*n* = 1132; 44.41%) with enteral nutrition being most commonly recommended (*n* = 509; 19.97%), followed by compensatory treatment (*n* = 396; 15.54%) and therapeutic treatment (*n* = 227; 8.91%) (Table 7). Language interventions were most frequently recommended for patients with CeVD (*n* = 645; 48.24%) followed by dysphagia interventions (*n* = 616; 46.07%) (Table 7).

**TABLE 7:** Speech-language therapy treatment recommendations.

SLT treatment recommendation	Recommendations: all patients (*n* = 2549)		Recommendations: patients with cerebrovascular disease (*n* = 1337)	
	*n*†	%	*n*†	%
1. Swallowing interventions:	1132	44.41	616	46.07
Enteral nutrition	509	19.97	269	20.12
Compensatory treatment	396	15.54	189	14.14
Therapeutic treatment	227	8.91	158	11.82
2. Speech interventions:	962	37.74	542	40.54
Dysarthria treatment	541	21.22	406	30.37
Apraxia treatment	168	6.59	123	9.20
Voice treatment	154	6.04	8	0.60
Burns: contracture prevention	93	3.65	1	0.07
Fluency treatment	6	0.24	4	0.30
3. Language interventions:	926	36.33	645	48.24
Multimodal treatment	503	19.73	362	27.08
Language treatment	263	10.32	207	15.48
AAC	115	4.51	34	2.54
Right hemisphere language treatment	45	1.77	42	3.14
4. Other:	352	13.81	185	13.84
Carer counselling/training	202	7.92	94	7.03
Monitoring	150	5.88	91	6.81
5. None	347	13.61	118	8.83
6. Cognitive-communication interventions	186	7.30	61	4.56

SLT, speech-language therapy.

† , Due to more than one treatment recommendation made for some patients, the total number of recommendations is greater than the number of patients.

#### Status at end of service

The overall mortality rate was 15.61% (*n* = 398). Patients with CeVD presented with the highest mortality rate (*n* = 268; 20.05%), and most patients discharged from SLT services due to being in the end of life phase (*n* = 78; 5.83%) (Table 8). Of all patients, 39.98% (*n* = 1019) required further treatment following hospital discharge, with this being true for over half of the patients with CeVD (*n* = 693; 51.83%) and TBI (*n* = 166; 52.37%) (Table 8). Speech-language therapy services ended for 56.67% (*n* = 85) of all patients for whom monitoring was recommended (*n* = 150), because of death or end of life status, with a median of 6 days (IQR = 2–12) between initial SLT contact and end of SLT service for these patients (*n* = 520; 20.40%).

**TABLE 8:** Status at end of inpatient speech-language therapy service for the top five medical conditions.

Status at end of inpatient SLT service	Medical condition									
	Cerebrovascular disease (*n* = 1337)		Traumatic brain injury (*n* = 317)		Other neurological (*n* = 281)		Cancer (*n* = 182)		Burns (*n* = 145)	
	*n*	%	*n*	%	*n*	%	*N*	%	*n*	%
Discharge: recovered function	152	11.37	74	23.34	58	20.64	27	14.84	65	44.83
End of life†	78	5.83	10	3.15	13	4.63	13	7.14	0	0.00
SLT management not further indicated‡	109	8.15	35	11.04	90	32.03	67	36.81	17	11.72
Continue SLT treatment post-hospital discharge	693	51.83	166	52.37	60	21.35	46	25.28	39	26.90
Deceased	268	20.05	23	7.26	43	15.30	11	6.04	19	13.10
Other§	37	2.77	9	2.84	17	6.05	18	9.89	5	3.45

SLT, speech-language therapy.

† , patients considered to be in the last weeks or days of life;

‡ , Due to medical condition (e.g. recommendation made for oesophageal dysphagia case, but further active SLT management not indicated);

§ , status at end of inpatient service not clearly recorded.

## Discussion

Most patients managed by SLTs presented with NCDs, followed by communicable diseases and injuries. This finding reflects the national shift from a historically higher prevalence of communicable diseases to the heavier burden of NCDs (Arokiasamy et al., 2015; Folb et al., 2015). This shift has been attributed in the literature to factors including low socio-economic status, low levels of education and income and unemployment (Arokiasamy et al., 2015), which describe many of the 83.6% of the public healthcare users in South Africa. Due to HIV status being documented as unknown for as many as 45% of patients, no conclusive links could be made between HIV and CeVD. Almost a third of patients reflected a complex profile of multimorbidity, which may have contributed to the high mortality rate of the caseload, and the large number requiring further SLT services post-hospital discharge. The latter emphasises the need for stepdown rehabilitation facilities and the provision of outpatient SLT services for optimisation of rehabilitation outcomes (Whitehead & Baalbergen, 2019).

The five most frequent medical conditions accounted for 88.74% of the caseload, which was led by CeVD (*n* = 1337; 52.45%), followed by TBI (*n* = 317; 12.44%), other neurological conditions (*n* = 281; 11.02%), cancer (*n* = 182; 7.14%) and burns (*n* = 145; 5.69%). Historically, CeVD has been associated with the elderly; however, with over a third of patients with CeVD being younger than 55 years, the rising trend of CeVD amongst younger adults was illustrated (Feigin et al., 2017; Onaolapo et al., 2019). This finding necessitates strong national preventative action and inclusion of return to work as a goal for SLT rehabilitation. The well-established correlation between hypertension and CeVD (Namale et al., 2018) was supported, indicating that hypertension may increase the risk of CeVD almost fivefold. Our finding, in light of the facts that 45% of South Africans aged 15 years and older are hypertensive (STATS SA, 2017b), and 91.1% are not screened, diagnosed, treated or controlled (Berry et al., 2017), speaks to the urgent need for increased awareness and control of hypertension (Arokiasamy et al., 2015; Berry et al., 2017).

The mortality rate for CeVD in the current study was 20.05% and could have been as high as 25.88% if those considered to be at the end of life were included. These rates are markedly higher than the global trend, which report inpatient mortality rates for CeVD to range from 9.46% to 11.60% (Cleveland Clinic: Neurological Institute Outcomes, 2018; Roberts, Thorne, Akbari, Samuel, & Williams, 2015). This finding could be related to South Africa’s complex BoD picture and may explain why fewer patients with CeVD in this study required continued treatment following hospital discharge as compared with two-thirds of patients with CeVD suggested globally (Winstein et al., 2016).

Burn injuries, which were among the top five medical conditions seen by SLTs in this study, are associated with orofacial and inhalation burns, the presence of artificial airway devices and prolonged mechanical ventilation, all of which may contribute to dysphagia and voice pathologies (Pavez & Martínez, 2019). For patients with acute burn injuries, SLTs are responsible for the prevention of orofacial contractures, tracheostomy management and management of dysphagia and communication disorders; however, SLTs are not yet widely accepted as a core part of the interdisciplinary team working in this context (Pavez & Martínez, 2019). This study advocates for training on the emerging role of the SLT in the assessment and management of patients with burn injuries, particularly given the urgency to intervene to prevent contractures, with consideration of medical factors such as pain management, wound dressings and the high risk of infection.

Dysphagia was the most prevalent SLT diagnosis, followed by aphasia and dysarthria. Almost 45% of the patients presented with multiple co-existing SLT diagnoses. This complexity correlated significantly with an increased number of SLT sessions and length of service. In the United States, acute care length of hospital stay ranges from 4 to 11 days (ASHA, 2019; Roberts et al., 2015; Winstein et al., 2016). While the true length of hospital stay could not be determined from the data in the study, there was a median of 13 days from hospital admission to the end of SLT service. It is suggested, then, that this was the minimum average length of hospital stay, which is higher than international findings described. Like other studies that reported no correlation between multimorbidity and length of hospital stay (Hewitt et al., 2016; Roche & De Vries, 2017), multimorbidity of medical conditions was not associated with a longer duration of SLT service in this study.

Doctors, typically the primary case contacts, were responsible for most referrals, while nurses, who have daily case contact and who are optimally positioned to detect communication and swallowing difficulties (Dondorf, Fabus, & Ghassemi, 2016), referred fewer than 1% of patients. This highlights the need for routine in-service training of nursing staff by SLTs regarding signs and symptoms of speech, language and swallowing disorders to affect timely and appropriate referrals (Dondorf et al., 2016). The quarter of case referrals from rehabilitation professionals reinforces the benefits of healthy working relationships, collaboration and awareness of roles within a multidisciplinary team (Dondorf et al., 2016; McGinnis et al., 2019; Winstein et al., 2016).

Early referral for rehabilitation is beneficial for outcomes across a variety of medical conditions (Pietsch, Lyon, & Dhillon, 2018; Winstein et al., 2016). In this study, the relationship between the number of days from admission to referral and the number of SLT sessions was not significant. However, it is well known that public-sector hospitals in South Africa are challenged by limited physical and human resources (Andrews & Pillay, 2017; Coutts, 2019), and hospital capacity and pressure for beds have both been documented as reasons for premature hospital discharge locally and internationally (Dreyer & Viljoen, 2019; Glette, Kringeland, Røise, & Wiig, 2019). For this reason, the possibility of earlier hospital discharge based on medical stability, regardless of the need for continued inpatient rehabilitation, should not be excluded.

Dysphagia influences not only length of hospital stay and cost of care (McGinnis et al., 2019) but also patient quality of life, nutrition, hydration, prolonged disability and even death (Andrews & Pillay, 2017; Jones, Cartwright, Whitworth, & Cocks, 2018; Pietsch et al., 2018). Often, compensatory treatment, such as texture modification or positioning, is recommended first because of the immediate impact on swallowing safety (Jones et al., 2018). However, therapeutic dysphagia intervention, such as manoeuvres or exercises, has been found to improve outcomes and reduce morbidity and mortality (Jones et al., 2018). It is thus concerning that for patients with dysphagia, therapeutic interventions were relatively infrequently recommended, and that dysphagia-specific interventions were not recommended at all for at least 9.29% of patients with dysphagia. Studies have found that newly graduated SLTs in South Africa lack confidence and feel unprepared with regard to dysphagia management (Singh et al., 2015; Yiannopoulos, 2016, as cited in Coutts, 2019), which may underlie a more conservative approach to dysphagia management in recommending enteral nutrition over other interventions. This likely impacts on outcomes and quality of life of patients presenting with dysphagia and suggests a need for further undergraduate training on the management thereof, as well as access to clinical supervision and evaluation in this area of practice.

Monitoring was recommended for 5.88% of patients, 56.67% of whom died or were at the end of life. These patients received SLT services for about 6 days, although it was unclear from the data captured what specific services were provided during this time. As many of these would have been in the pre-active and active phases of dying (Toner & Shadden, 2012), the SLT’s role in palliative and end-of-life care should be considered. Although there is clearly a role for SLTs in this context, there do not appear to be clear guidelines for practice (Chahda, Mathisen, & Carey, 2017; Toner & Shadden, 2012). As urged by Chahda et al. (2017), goals were adjusted at the study institution following this research to focus on quality of life rather than on rehabilitation. There is an urgent need for guidelines on the emerging role of SLTs in South Africa working in this emotionally, physically and ethically charged area (Toner & Shadden, 2012).

## Conclusion

While a large data set was analysed, only a few key findings could be discussed in this article. Speech-language therapy patients in South Africa reflect the country’s heavy burden of NCDs and multimorbidity. There was evidence of CeVDs in younger adults under 55 years of age, as well as a high proportion of patients who presented with more than one SLT diagnosis (44.68%) and consequent increased duration of service. These findings highlight the need to optimise human resources, identify appropriate candidates for SLT services and outline suitable packages of care for inpatients. The high prevalence of dysphagia also requires both knowledge and skill to optimise outcomes, which places training demands, both in theory and practice, on higher education institutions and advocates for improved clinical supervision at hospital level, especially considering the profile of increased case complexity evidenced. Finally, the ever-evolving profile of patients seen by SLTs in the public health sector in South Africa should continually be explored.

## Limitations

The institution’s protocol dictated that SLTs attend to all referrals made for services. This led to the data of some patients being captured despite, for instance, being inappropriate because of medical conditions (e.g. delirium because of renal failure) or having intact function. While this allows for broader insights into referral patterns and caseloads, it also suggests that the multidisciplinary care pathway into SLT services could yet be refined. When translating the documented medical condition from the official hospital records into the original electronic database, inconsistencies may have occurred, resulting in an overrepresentation of, for example, conditions classified as other neurological, where another diagnosis may have been more accurate. There may also have been discrepancies in the interpretation of variables, for instance, ending SLT services because of intact function versus management not being further indicated. This could account for the lower inter-rater agreement for the status at the end of SLT service. Additionally, conclusions regarding specific treatments provided, patient outcomes and length of hospital stay could not be drawn because of non-availability of relevant data. Future studies should explore patient outcomes, specifically as they relate to BoD, SLT diagnoses and the specific nature of interventions.
